# A Rare Case of Non-Small Cell Lung Cancer Presenting as Exudative Retinal Detachment

**DOI:** 10.5334/jbsr.3533

**Published:** 2024-03-19

**Authors:** Raphaëlle Dermine, Pierre-Antoine Poncelet, Vincent Verschaeve

**Affiliations:** 1 Physician Assistant, Cliniques Universitaires Saint-Luc UCLouvain, Bruxelles; 2 Head of Radiology Department, Grand Hôpital de Charleroi; 3 Oncologist, Grand Hôpital de Charleroi

**Keywords:** Non-small cell lung cancer, Metastasis, Retinal detachment, Orbital metastases

## Abstract

*Teaching Point:* Retinal detachment is a rare initial clinical manifestation of lung cancer with intraorbital metastases, early diagnosis on magnetic resonance imaging is important for therapeutic implications.

## Case History

A 61-year-old woman presented to the emergency department with a 2-week history of gradual deteriorating vision, morning headache, and nausea. She complained about a severe painless loss of vision in her right eye with blurred vision. Clinical examination revealed a decreased breath sound in the mid- to left-lower lung zones and instability on walking. An ophthalmic examination of the right eye showed a decreasing visual acuity. Fundus examination demonstrated right-sided exudative retinal detachment.

**Figure 1:**
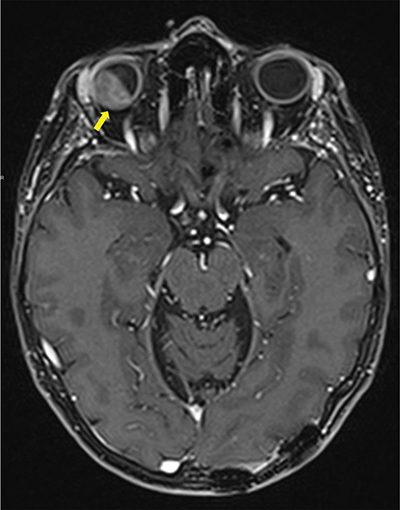
Contrast enhanced Axial T1 Dixon MRI-scan showing intra-orbital metastasis (arrow) invading all layers of the eyeball and the intra-conal fat.

**Figure 2:**
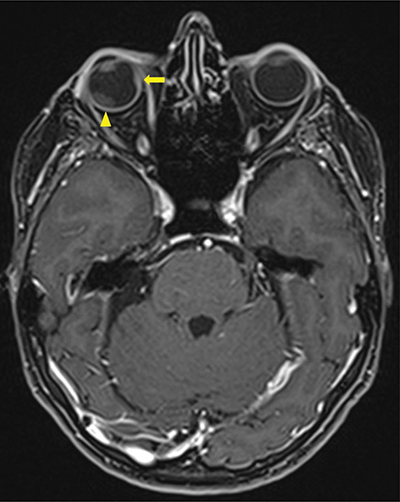
Contrast enhanced Axial T1 Dixon MRI-scan showing a second intra-orbital metastasis (arrow) invading all layers of the eyeball including sclera and a secondary retinal detachment (arrowhead).

**Figure 3:**
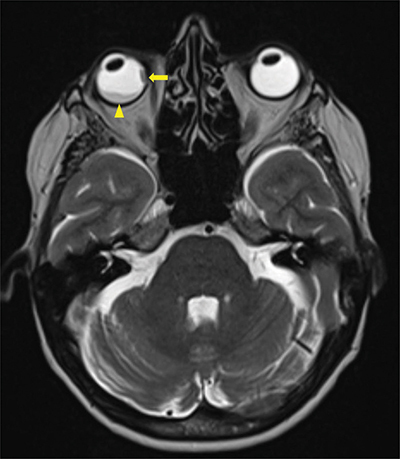
Axial T2 Dixon MRI-scan showing a second intra-orbital metastasis (arrow) invading all layers of the eyeball including sclera and a secondary retinal detachment (arrowhead).

A magnetic resonance image (MRI) demonstrated a 16.5 × 10.7 mm right intraorbital mass of superolateral topography invading all of the ocular globe layers (Figure 1), and a second medial 8 × 3.7 mm right intraorbital mass (arrow, Figure 2 T1, Figure 3 T2). The posterior sclera and the periorbital fat were invaded, with partial right retinal detachment (arrowhead, Figure 2 T1**,** Figure 3 T2).

These ocular lesions were associated with two left posterior parietal and left occipital necrotic-hemorrhagic lesions. Screening examinations were carried out as these lesions were suspected of being metastases. Chest computer tomography revealed a large mass on the left upper lung lobe and a small nodule on the left lower lobe suggestive of metastasis.

Biopsy results from bronchoscopy identified poorly differentiated non-small-cell lung carcinoma.

Pathological results from the excision of both cerebral lesions were compatible with a primitive lung origin.

## Comments

Here we present a rare case of a patient with orbital metastases from non-small cell lung carcinoma, with ocular symptoms preceding the diagnosis of neoplastic process. The initial manifestation of the disease was the choroidal mass with secondary retinal detachment.

NSCLC metastases to the choroid are uncommon, only a few reports exist about retinal detachment due to intraorbital metastases.

Based on previous reports [1], breast and lung tumors are the most common primary tumors causing intraorbital metastases. Despite being rare, symptoms of the orbital (blurred vision or changes in visual acuity) may be early clinical manifestations, before the diagnosis of a lung tumor is established.

MRI is an appropriate investigation in diagnosing orbital lesions, showing higher soft tissue contrast.

Orbital metastases are generally associated with a poor systemic prognosis, indicating the spread of hematogenous cancer and a late stage of the lung carcinoma, with an average survival estimated to be no longer than 5 to 6 months.

In this case, our patient received radiotherapy associated with first-line chemo-immunotherapy. Systemic corticotherapy was given transitory to reduce the inflammatory component of exudative retinal detachment. Being progressive after two courses, with the appearance of liver metastases, palliative care has been decided without MRI control. However, the orbital metastases responded well clinically to radiotherapy.
